# Supplementary material for: Imaging-Based Characterization of a Slco2b1(-/-) Mouse Model Using [11C]Erlotinib and [99mTc]Mebrofenin as Probe Substrates
Source: Pharmaceutics. 2021 Jun 21;13(6):918. doi: 10.3390/pharmaceutics13060918 (PMC8233734; doi:10.3390/pharmaceutics13060918)
Supplement: Supplementary file 1 [file pharmaceutics-13-00918-s001.zip › pharmaceutics-1236063- for conv supp.pdf]

pharmaceutics

MDPI

Supplementary Materials: Imaging-Based Characterization of a *Slco2b1*$^{(-/-)}$ Mouse Model Using [^{11}C]erlotinib and [^{99m}Tc]mebrofenin as Probe Substrates

Solène Marie, Irene Hernández-Lozano, Louise Breuil, Charles Truillet, Shuiying Hu, Alex Sparreboom, Nicolas Tournier and Oliver Langer

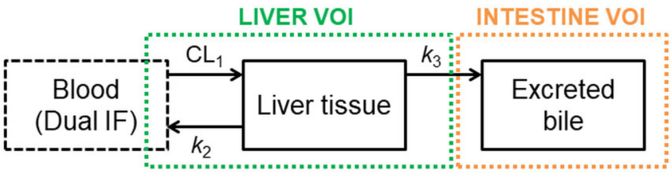

Figure S1. Pharmacokinetic model implemented to assess the hepatobiliary disposition of [^{11}C]erlotinib and [^{99m}Tc]mebrofenin. CL_1 (mL/min) represents the hepatic uptake clearance, and k_2 and k_3 (min^{-1}) are the rate constants defining the transfer of radioactivity from liver to blood and from liver to intestine, respectively. The model was modified from a previously published liver model [15]. IF, input function; VOI, volume of interest.

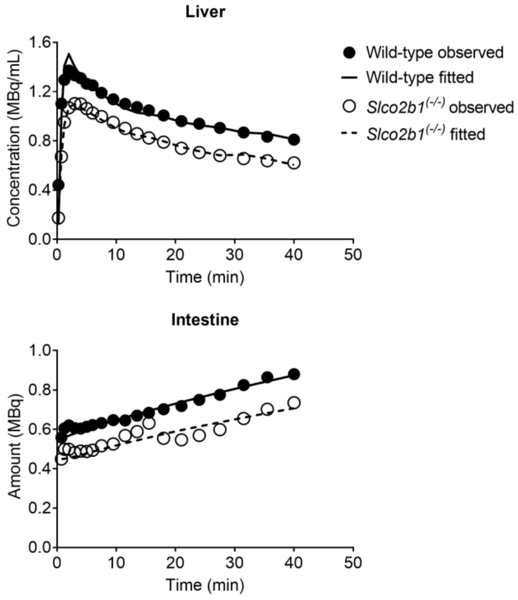

Figure S2. Observed and fitted time-activity curves (MBq/mL or MBq) of [^{11}C]erlotinib in the liver and intestine in one representative wild-type and *Slco2b1*$^{(-/-)}$ mouse.

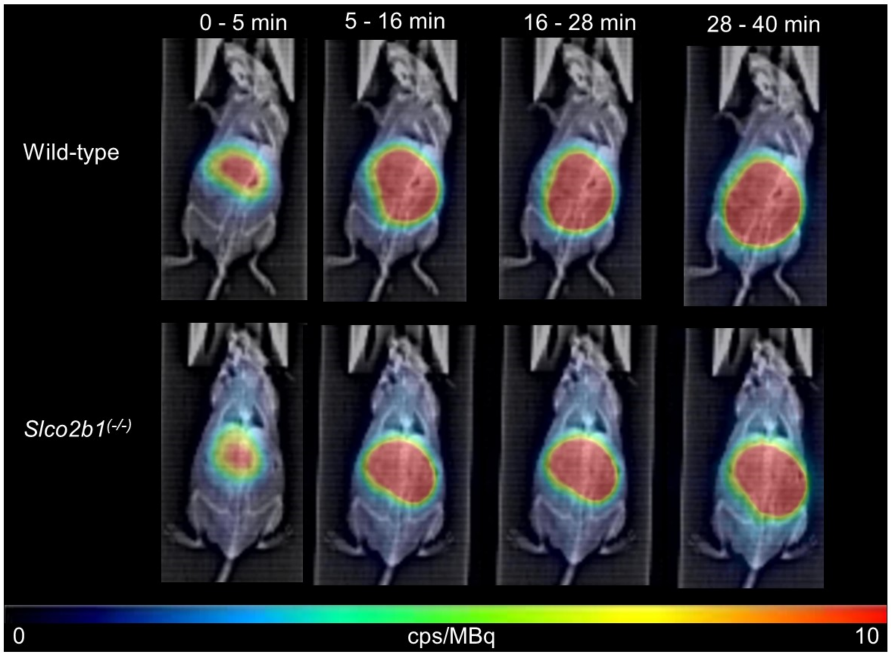

Figure S3. Serial CT-co-registered planar scintigraphy images of one representative wild-type and *Slco2b1*$^{(-/-)}$ mouse after i.v. injection of [^{99m}Tc]mebrofenin. Radioactivity counts (counts per second, cps) are normalized to the injected radioactivity amount (cps/MBq).

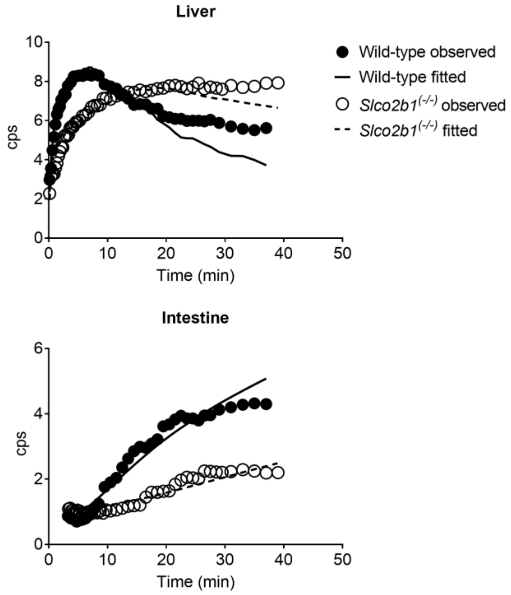

Figure S4. Observed and fitted time-activity curves (cps) of [^{99m}Tc]mebrofenin in the liver and intestine in one representative wild-type and *Slco2b1*$^{(-/-)}$ mouse.
